# Antibacterial Activity of Fusidic Acid-Loaded Electrospun Polylactide Fiber Fleeces Against Periodontopathogenic Species

**DOI:** 10.3390/pharmaceutics17070821

**Published:** 2025-06-24

**Authors:** Bernd W. Sigusch, Markus Reise, Stefan Kranz, Julius Beck, Kerstin Wagner, André Guellmar, Markus Heyder

**Affiliations:** 1 Department of Conservative Dentistry and Periodontology, Center of Dental Medicine, Jena University Hospitals, 07743 Jena, Germany; bernd.w.sigusch@med.uni-jena.de (B.W.S.); markus.reise@med.uni-jena.de (M.R.); julius.beck@med.uni-jena.de (J.B.); andre.guellmar@med.uni-jena.de (A.G.); markus.heyder@med.uni-jena.de (M.H.); 2 Innovent e.V., 07745 Jena, Germany; k.wagner@innovent-jena.de

**Keywords:** antibiotics, periodontitis, *Porphyromonas gingivalis*, local drug delivery, *Staphylococcus aureus*

## Abstract

**Background/Objectives:** The effect of fusidic acid on oral bacteria, especially on Gram- negative periodontopathogenic species, has not yet been investigated. This in vitro study aimed to analyze the antibacterial effect of fusidic acid alone and as an active component in electrospun poly(L-lactide-co-D/L-lactide) fiber fleeces. **Methods:** Minimal inhibitory concentrations (MIC) of fusidic acid and metronidazole (control) were determined for various oral bacteria. Eluates were collected from electrospun poly(L-lactide-co-D/L-lactide) fiber fleeces loaded with 10 and 20 wt% fusidic acid over a period of 28 d. Antibacterial activity was analyzed by means of a microdilution assay. Cytotoxicity was observed toward human gingival fibroblasts (HGFs). **Results:** All tested Gram-positive and Gram-negative oral bacteria were susceptible to fusidic acid. The lowest MIC was observed for *Porphyromonas gingivalis* (MIC < 0.062 µg/mL). Compared to the antibacterial activity of metronidazole, that of *Porphyromonas gingivalis* was suppressed by significant lower fusidic acid concentrations (*p* < 0.01). The eluates obtained from electrospun poly(L-lactide-co-D/L-lactide) fiber fleeces inhibited the growth of *P. gingivalis*, *S. aureus*, *A. viscosus*, and *A. neslundii* over a course of 28 days. The largest inhibition zones were detected for *Porphyromonas gingivalis* in case of the 20 wt% concentrations. The eluates were not cytotoxic toward HGFs. **Conclusions:** It was shown that fusidic acid has significant antibacterial potential. The results of the present investigation suggest that fusidic acid alone or delivered by electrospun fiber fleeces might be attractive for controlling oral pathogenic bacteria.

## 1. Introduction

Periodontitis is a chronic inflammatory disease that is characterized by a gradual loss of tooth supporting tissues. The disease arises from a dysbiotic shift in the oral microbial community, which elicits and maintains a self-destructive host immune response [1,2]. Clinically, periodontitis manifests in the formation of periodontal pockets, gingival recessions, and increased tooth mobility. In order to prevent further tissue damage, initial treatment mainly aims at the removal and destruction of microbial biofilms. This is accomplished by careful scaling and planing (SRP) of the root surface in conjunction with local supportive antimicrobial measures and, in severe cases, additional administration of antibiotics [3,4,5,6].

The range of antimicrobials used to treat periodontitis is rather extensive, and their effectiveness varies considerably depending on the agent and protocol applied. Numerous randomized clinical trials and systematic reviews have shown benefits from the use of antimicrobials over mechanical treatment alone [7,8,9,10]. Especially in severe cases, systemic antibiosis with metronidazole and amoxicillin has shown significant impact on pocket depth reduction and clinical attachment gain [8,9].

For local periodontitis treatment, the delivery of anti-infective agents by electrospun fiber fleeces is of potential interest [11,12]. In this regard, electrospun fibers are considered efficient carrier systems, especially for the local application of antimicrobials [11,12,13,14,15,16].

In addition to their drug carrier function, electrospun fibers are also used in various non-invasive medical applications. These include biosensors, personal protection equipment, and wound dressings. Furthermore, surface modification strategies even enable adaptation to many other approaches in the biomedical field [17,18].

However, some of these fiber systems are still afflicted by a rather strong initial release of the incorporated drug, which has also been acknowledged by our own research group in previous studies [12,19]. This so called initial burst limits the long-term efficiency and has a distinct impact on the desired medical outcome [13,20]. In this regard, our group has proven that coaxial electrospun fibers with a core–shell design follow a more constant release kinetic with less signs of an initial burst [11].

**Figure 1:**
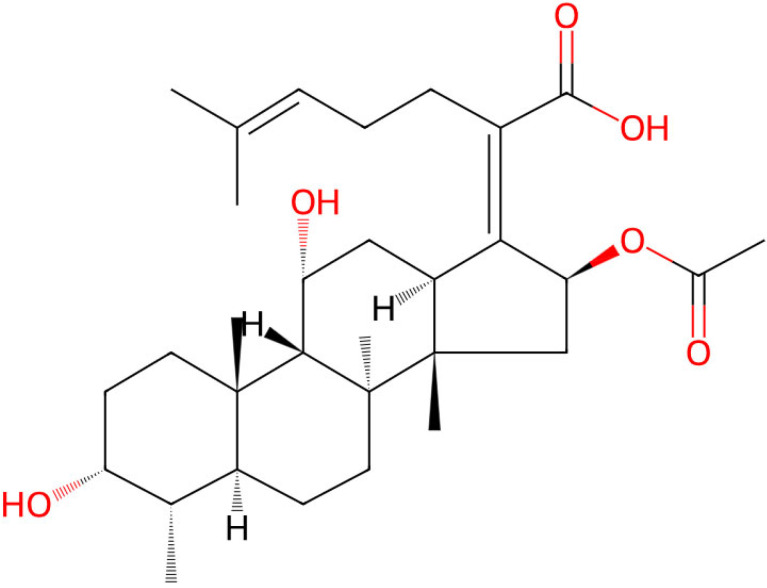
Chemical structure of fusidic acid. Source: https://www.chemspider.com.

In the local treatment of periodontitis there is still high demand for efficient antimicrobials. Therefore, this study aimed to analyze the susceptibility of various pathogenic oral bacteria toward fusidic acid. Fusidic acid (Figure 1) belongs to the class of antibiotics with activity mainly against Gram-positive bacteria, such as coagulase-negative Staphylococci, beta-hemolytic Streptococcus, Corynebacteria, and Clostridia [21,22]. Some information about borderline activity against anaerobic Gram-negative rods is already available, with resistance being demonstrated for most Fusobacterium species [23]. The efficiency of fusidic acid in suppressing oral bacteria, especially Gram-negative periodontopathogenic species such as *F. nucleatum*, *A. actinomycetemcomitans*, and *P. gingivalis*, has not yet been investigated in detail.

However, fusidic acid is commonly applied in dermatology and ophthalmology, especially for the topical cure of skin and soft tissue infections that are caused by *staphylococci* [24,25,26,27]. The agent is also used in combination with other antimicrobials (e.g., rifampin) in the treatment of topical infections with MRSA [22].

So far, information about the antibacterial effect of fusidic acid on oral bacteria, especially on Gram-negative periodontopathogenic species, is limited. Therefore, the present investigation aimed to evaluate the susceptibility of various Gram-positive and Gram-negative oral pathogenic bacteria toward fusidic acid. Furthermore, electrospun poly(L-lactide-co-D/L-lactide) fiber fleeces loaded with fusidic acid were analyzed for their antibacterial and cytocompatible characteristics. Hence, it should be determined if fusidic acid-loaded fiber fleeces are efficient in controlling oral bacteria in vitro.

## 2. Materials and Methods

### 2.1. Bacterial Species

The antibacterial effect of fusidic acid was investigated on the following bacterial species: *Actinomyces viscosus* (DSMZ 43327/ATCC 15987), *Actinomyces naeslundi* (DSMZ 17233/ATCC 19039), *Streptococcus gordonii* (DSMZ 20568/ATCC 33399), *Streptococcus sobrinus* (DSMZ 20742/ATCC 33478), *Streptococcus mutans* (DSMZ 20523/ATCC 25175), *Enterococcus faecalis* (DSMZ 20478/ATCC 19433), *Staphylococcus aureus* (ATCC 49230), *Fusobacterium nucleatum* (DSMZ 20482/ATCC 10953), *Aggregatibacter actinomycetemcommitans* (DSMZ 11123/ATCC 43718), and *Porphyromonas gingivalis* (ATCC 53978).

At first, bacteria were grown in their respective culture medium. Schaedler fluid medium supplemented with vitamin K was used for Actinomycetes spp., *F. nucleatum*, *A. actinomycetemcommitans*, *P. gingivalis*, and *E. faecalis*. The oral streptococci as well as *S. aureus* were grown in tryptone-soy nutrient medium. All batches were incubated at 37 °C under anaerobic standard conditions for 24 h.

Subsequently, bacteria were pelleted by centrifugation, washed twice with PBS, and resuspended in the respective nutritious medium at an OD_546nm_ of 0.1. This correlates with a bacterial concentration of 1 × 10^7^ CFU/mL.

### 2.2. Microdilution Assay

The Boullion microdilution test was applied to assess the minimal inhibitory concentration (MIC) of fusidic acid. The arranged bacterial suspensions were diluted to receive a bacterial concentration of 1 × 10^6^ CFU/mL.

For the investigation, 100 μL of the specific culture medium and a 1:2 dilution of fusidic acid in concentrations ranging between 256 μg/mL and 0.125 μg/mL were pipetted into 96-well microtitration plates. Next, 100 μL of the respective bacterial suspension was added to each well, which resulted in effective drug concentrations ranging between 128 μg/mL and 0.062 μg/mL. Analogously, for all tested pathogens, a metronidazole dilution series was arranged, which served as control. On each microtiter plate, 6 additional wells were used as negative controls. Each of these wells was filled with 100 μL of the respective nutrient medium without fusidic acid or metronidazole and with 100 μL of the respective bacterial solution. Subsequently, all plates were incubated for 24 h at 37 °C under strain-specific conditions.

The bacterial growth was assessed both visually and photometrically using a Lambda Scan 200 spectrophotometer and the KC4 program at 546 nm. The lowest drug concentrations at which the solutions did not become turbid corresponded to the minimum inhibitory concentration (MIC). For each drug concentration, 6 test batches were arranged. Six additional batches served as controls.

### 2.3. Manufacturing of Electrospun Poly(L-Lactide-Co-D/L-Lactide) Fiber Fleeces Loaded with Fusidic Acid

For the electrospinning process, a solution of 3–5% poly(L-lactide-co-D/L-lactide) (70:30, Resomer^®^ LR708, Evonik, Essen, Germany) and fusidic acid sodium salt (10, 20 wt%) was used. The spinning process was conducted at 20–24 kV with a flow rate of 2.0 mL/h. This enabled the fabrication of fiber fleeces with fiber diameters ranging between 1 and 3 µm.

In total, 24 samples, 12 with 10 wt% fusidic acid sodium salt and a mean weight each of 2.6 mg (±0.2 mg) and 12 samples with 20 wt% fusidic acid with a mean weight of 4.5 mg (±0.4 mg), were fabricated.

### 2.4. Optical Characterization of the Electrospun Poly(L-Lactide-Co-D/L-Lactide) Fiber Fleeces

After electrospinning, fiber fleeces with and without fusidic acid were optically characterized using an Axiotech microscope (Zeiss, Oberkochen, Germany). The mean fiber diameters were obtained and representative pictures were taken.

### 2.5. Collection of Eluates for Antibacterial and Cytocompatibility Assessment

In order to assess the antibacterial activity and cytotoxic behavior of the manufactured fusidic acid-loaded fiber fleeces, eluates were obtained at defined time points. In total, 12 samples (1.5 mm in diameter), each with either 10 wt% or 20 wt% fusidic acid concentration, were placed in 12-well incubation plates and sterilized by UV radiation for 10 min. Half of the samples were then covered with 2 mL PBS to analyze the antibacterial effect. The remaining fleeces were placed in 2 mL DMEM supplemented with 10% fetal calf serum for cytocompatibility testing. All batches were incubated at 37 °C under a standard atmosphere. Eluates (2 mL) were collected after 0.5 h, 1 h, 3 h, 6 h, 12 h, 24 h, 48 h, 72 h, 96 h, 120 h, 144 h, 168 h, 336 h, 504 h, and 672 h. All collected samples were immediately frozen at −20 °C and stored until use.

### 2.6. Agar Diffusion Assay

The antibacterial activity of the obtained eluates was tested by means of an agar diffusion assay. For *Staphylococcus aureus* and all oral Streptococci spp., Petri dishes with tryptone soya agar were prepared. Actinomycetes spp., *F. nucleatum*, *A. actinomycetemcommitans*, *P. gingivalis*, and *E. faecalis* were tested on Schaedler agar supplemented with 1% vitamin K and 10% sheep blood. Bacterial suspensions were prepared according to the above scheme. Subsequently, aliquots (100 μL) of each suspension were spread on the previously prepared agar plates. Afterward, holes (8 mm in diameter) were punched and filled with the respective eluates. After incubation for 48 h, the diameters of the resulting inhibition zones were measured.

### 2.7. Cytotoxicity Assessment

Human gingival fibroblasts were grown in culture medium composed of 90% DMEM, 10% FBS, and 1% antibiotic/antimycotic solution (AAS) at 37 °C and under a 5% CO_2_ atmosphere. At the end of the 5th passage, cells were harvested and seeded (10^4^ cells/mL) in 96-well microtitration plates. Culture media without AAS was mixed with fusidic acid and metronidazole in concentrations ranging between 0.062 and 128 µg/mL and added to the wells. Cells incubated only with culture media served as controls. All test batches were incubated for 48 h at 37 °C under a standard atmosphere.

In a second setup, the cytotoxicity of the collected eluates obtained from fusidic acid-loaded fiber fleeces (10 and 20 wt%) was tested. Therefore, HGFs were seeded in 96-well microtitration plates (104 cells/mL) and the respective eluates were added. All batches were incubated for 48 h at 37 °C. Cell viability was evaluated by XTT-test.

### 2.8. Statistical Analysis

The statistical evaluation was carried out using SPSS 28.0 for Windows. A one factor analysis of variance was used to compare the means of the microdilution assay, which was adjusted using Welch’s test. Post hoc Bonferroni correction was applied. Significant differences between the various groups of the agar diffusion assay were determined using the Mann–Whitney U-test. Significance among the means of the cytotoxicity assay was determined by multi-variant, linear regression analysis. The level of significance was *p* < 0.05.

## 3. Results

### 3.1. Minimal Inhibitory Concentrations of Fusidic Acid for Various Oral Bacteria

**Figure 2:**
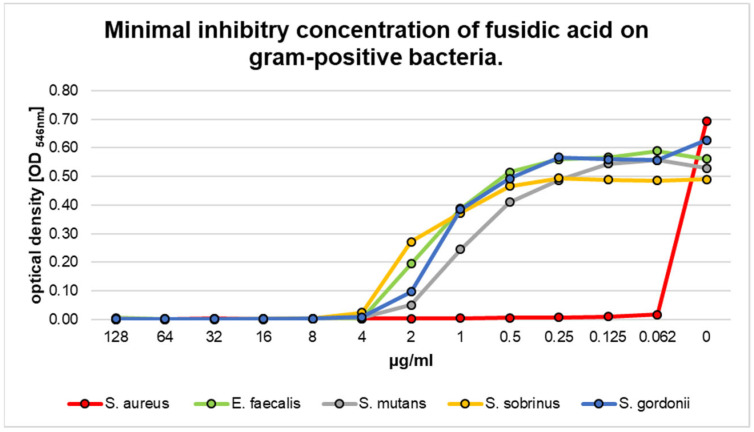
Minimal inhibitory concentration (MIC) of fusidic acid for different Gram-positive bacterial species.

The minimum inhibitory concentration (MIC) values of fusidic acid for the investigated Gram-positive species are summarized in Figure 2. The optical density (OD) was used to illustrate the inhibitory effects. The MIC is defined as lowest drug concentration at which visible growth is inhibited (OD = 0). For the Gram-positive species *S. mutans*, *S. sobrinus*, and *E. faecalis*, an MIC of 4 μg/mL was evaluated. The highest susceptibility toward fusidic acid was detected for *S. aureus*. For this species, an MIC of <0.062 μg/mL was determined (red graph, Figure 1), which was significantly different from all other Gram-positive bacteria (*p* < 0.01).

**Figure 3:**
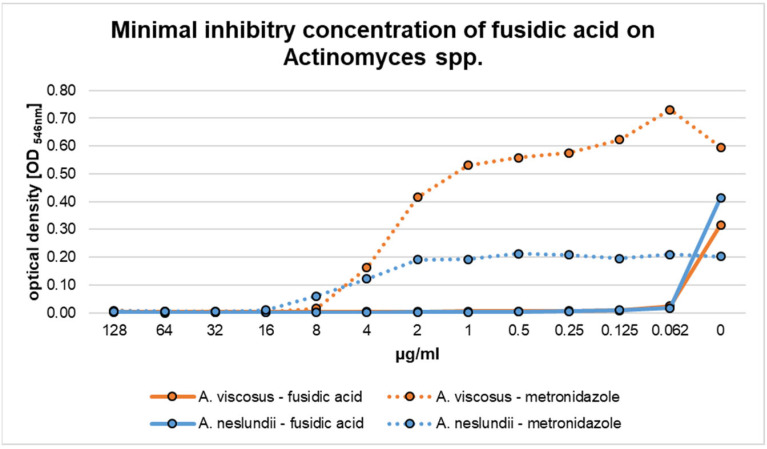
Minimal inhibitory concentration (MIC) of fusidic acid and metronidazole for *Actinomyces viscosus* and *Actinomyces neslundii*.

The results regarding the filamentous Gram-positive species *A. viscosus* and *A. neslundii* are summarized in Figure 3. Both species presented a high susceptibility toward fusidic acid. For both bacteria, an MIC of 0.062 µg/mL was obtained. In the case of metronidazole, higher MIC values were detected. In detail, treatment with metronidazole resulted in minimal inhibitory concentrations of 8 µg/mL and 16 μg/mL for *A. viscosus* and *A. neslundii*, respectively (Figure 2). There was a significant difference between both species when compared to the susceptibility toward fusidic acid (*p* < 0.01).

**Figure 4:**
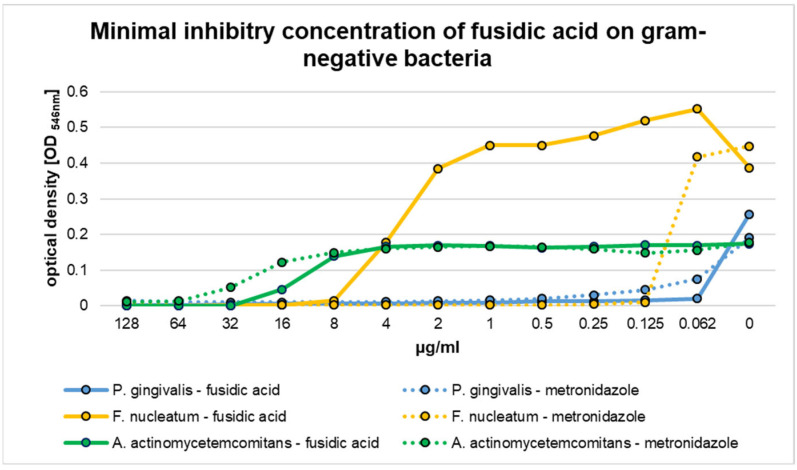
Minimal inhibitory concentration (MIC) of fusidic acid and metronidazole for Gram-negative *Porphyromonas gingivalis*, *Fusobacterium nucleatum*, and *Aggregatibacter actinomycetemcomitans*.

A high susceptibility toward fusidic acid was also observed for the Gram-negative species *P. gingivalis* (Figure 4). An MIC of <0.062 μg/mL fusidic acid was sufficient to prevent visible bacterial growth. By comparison, an MIC of 0.5 μg/mL was assessed for metronidazole. Both values did not significantly differ from each another (*p* = 1.0).

Gram-negative *F. nucleatum* was also sensitive toward fusidic acid. An MIC of 8 μg/mL resulted in sufficient visible growth inhibition. By comparison, metronidazole showed an MIC of 0.125 μg/mL for this species, which was significant different (*p* < 0.01).

The highest MIC of fusidic acid was observed for Gram-negative *A. actinomycetemcomitans* (Figure 3). For this species, an MIC of 32 μg/mL was obtained, which did not significantly differ from the value analyzed for metronidazole (64 μg/mL, *p* = 1.0).

In conclusion, all tested Gram-positive and Gram-negative species were susceptible toward fusidic acid. The lowest MIC values were obtained for the species *S. aureus*, *A. neslundii*, *A. viscosus*, and *P. gingivalis* (MIC < 0.062 µg/mL). Compared to the antibacterial activity of metronidazole, the growth of *A. neslundii*, *A. viscosus*, *P. gingivalis*, and *A. actinomycetemcomitans* was suppressed by lower fusidic acid concentrations. Only in the case of *F. nucleatum* did metronidazole show a significantly lower MIC.

### 3.2. Microscopic Characterization of Poly(L-Lactide-Co-D/L-Lactide) Fiber Fleeces

**Figure 5:**
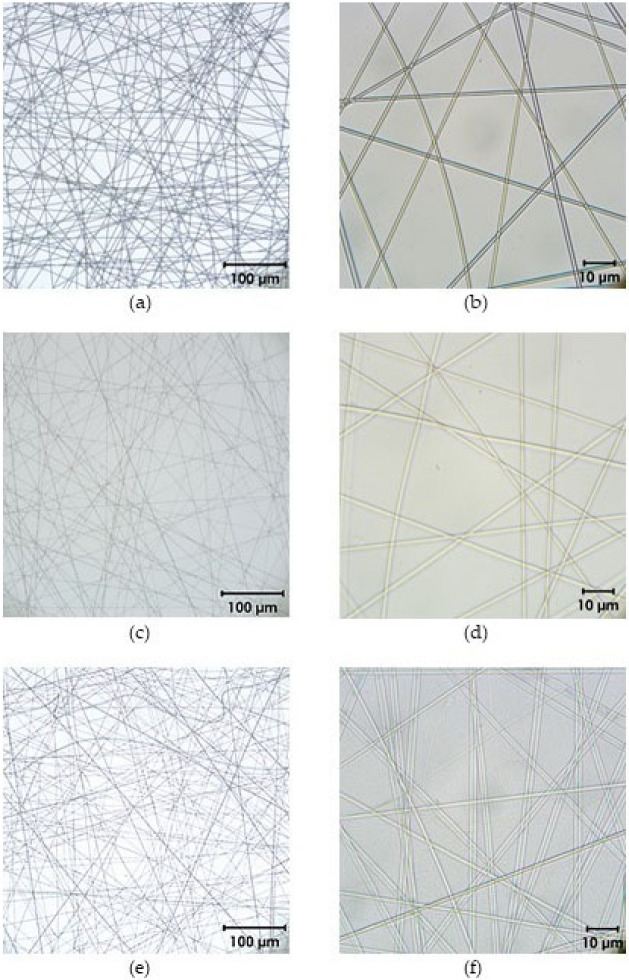
Microscopic characterization of fusidic acid-loaded electrospun fiber fleeces: (**a**,**b**) Fleeces without fusidic acid; (**c**,**d**) electrospun fiber fleeces with 10 wt% fusidic acid; (**e**,**f**) fiber fleeces with 20 wt% fusidic acid. Pictures are presented at magnifications of 20× (left) and 100× (right).

Representative pictures of the fabricated electrospun fiber fleeces are shown in Figure 5. All fibers had a homogeneous appearance, regardless of the incorporated fusidic acid concentration. The diameters were 1.1 ± 0.2 µm for fibers without fusidic acid. Fibers with 10 wt% fusidic acid showed diameters of 0.9 ± 0.1 µm, while for fibers with a 20 wt% fusidic acid concentration, diameters of 2.2 ± 0.4 µm were measured.

### 3.3. Antibacterial Effect of Fusidic Acid-Loaded Electrospun Fiber Fleeces—Agar Diffusion Assay

**Figure 6:**
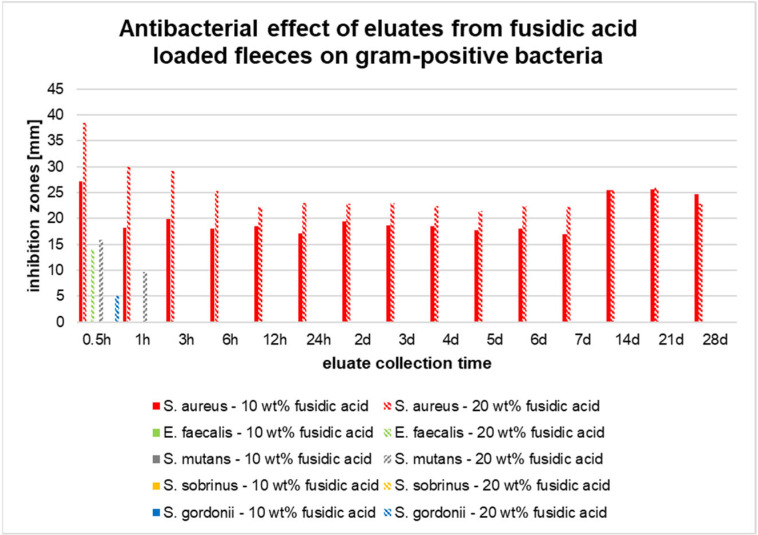
Agar diffusion assay. Inhibition zones of eluates collected from fusidic acid-loaded fiber fleeces (10 and 20 wt%) for different Gram-positive bacterial species over a course of 28 d.

The eluates obtained from the electrospun polylactide fleeces loaded with fusidic acid in concentrations of 10 and 20 wt% presented only minor effects on most of the tested Gram-positive cocci species, except *S. aureus* (Figure 6) showed a significant reduction. Inhibition zones were detected for *S. gordonii*, *S. mutans*, and *E. faecalis* only for the eluates obtained from the 20 wt% fleeces. In detail, inhibition zones for *S. gordonii* and *E. faecalis* were detected only for the eluates collected after 0.5 h of incubation. *S. mutans* additionally showed an inhibition zone after one hour of incubation. All of the other eluates collected from either the 10 or 20 wt% fleeces failed to induce any inhibition zones (Figure 6). No inhibition zones were found for the Gram-positive species *S. sobrinus*.

By contrast, *S. aureus* showed high susceptibility. The eluates from both fleece concentrations induced major inhibition zones at any observation point. Significant differences between both concentrations were estimated at all time points, except for the eluates collected after 2 d, 14 d, 21 d, and 28 d (Figure 6).

**Figure 7:**
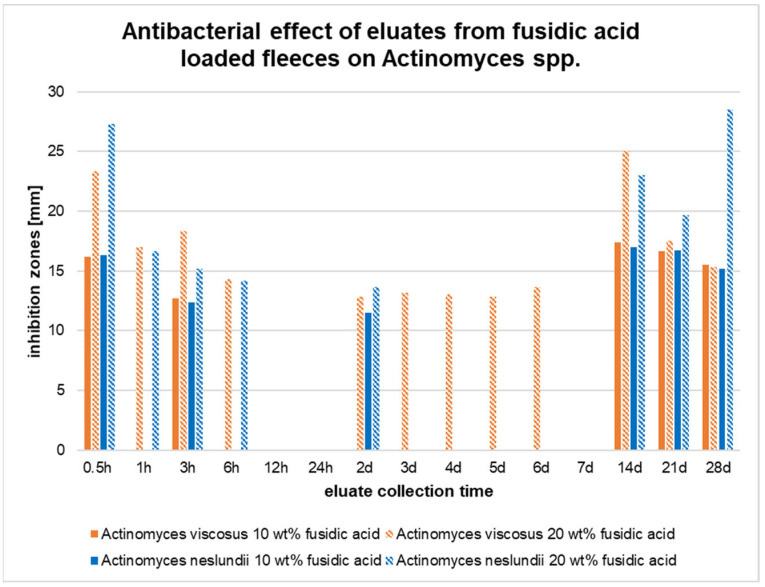
Results from the agar diffusion assay of eluates collected from fusidic acid-loaded fiber fleeces (10 and 20 wt%) for *Actinomyces viscosus* and *Actinomyces neslundii*.

The collected eluates also induced major inhibition zones for both Actinomyces spp. (Figure 7). Samples obtained from the 10 wt% fleeces caused inhibition zones for *A. viscosus* after 0.5 h, 3 h, 14 d, 21 d, and 28 d of incubation. The eluates collected from the 20 wt% fleeces additionally induced inhibition zones after 1 h and 6 h as well as between days 2 and 6.

Up to day 14, the inhibition zones were significantly larger compared to those produced by the 10 wt% samples (*p* < 0.01). All other inhibition zones did not significantly differ in size from each another (Figure 7).

The species *A. naeslundii* was also inhibited by the eluates collected from the 10 wt% fusidic acid-loaded fleeces after 0.5 h and 3 h and at days 2, 14, 21, and 28 of incubation. The eluates from the 20 wt% fusidic acid-loaded fleeces induced inhibition zones after 1 h, 6 h, and after 6 days of incubation. The inhibition zones of these eluates were at all times significantly larger compared to those obtained from the eluates collected from the 10 wt% fusidic acid-loaded fleeces (Figure 7).

**Figure 8:**
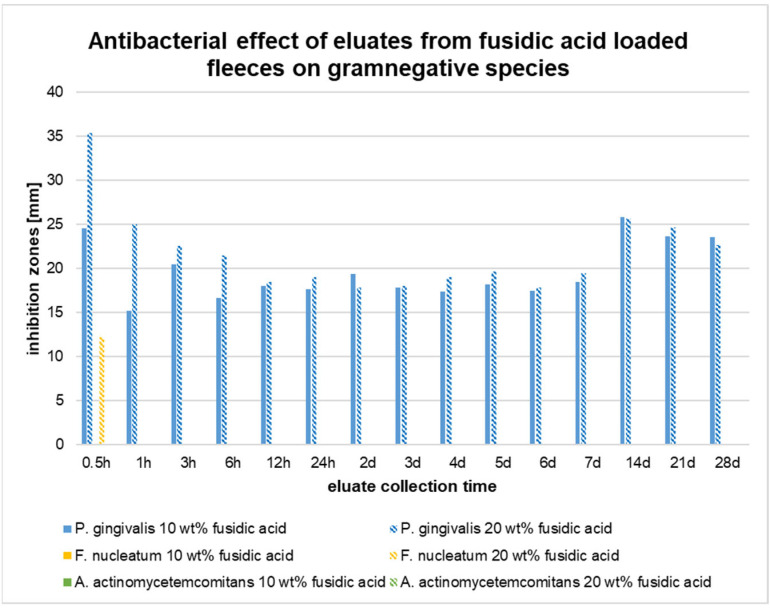
Agar diffusion assay of eluates collected from fiber fleeces loaded with 10 and 20 wt% fusidic acid for different Gram-negative bacterial species over a study period of 28 d.

Similar to the oral Gram-positive cocci species, almost no inhibitory effects were observed for Gram-negative *F. nucelatum* and *A. actinomycetemcomitans* (Figure 8). Only eluates collected from the 20 wt% fleeces after 0.5 h of incubation induced inhibition zones for *F. nucleatum*. In the case of *A. actinomycetemcomitans*, none of the collected eluates showed any inhibitory effect in the agar diffusion assays.

By contrast, *P. gingivalis* showed high susceptibility. The eluates collected from the 10 and 20 wt% fleeces induced major inhibition zones at any time point. The eluates collected from the 20 wt% fleeces after 0.5 h, 1 h, 6 h and 4 d resulted in significantly larger inhibition zones as compared to the 10 wt% eluates (Figure 8).

In conclusion, the eluates obtained from fiber fleeces with 10 and 20 wt% fusidic acid induced major inhibition zones for the species *S. aureus*, *A. neslundii*, *A. viscosus*, and *P. gingivalis*. No inhibition zones were observed for the species *S. sobrinus* and *A. actinomycetemcomitans*. The eluates obtained from the 20 wt% fleeces showed only minor inhibitory effects on *S. mutans*, *S. gordonii*, and *E. faecalis*. The eluates from the 10 wt% fleeces totally failed to inhibit these species.

## 4. Discussion

In the present study, the antibacterial effect of fusidic acid alone or as an active ingredient in drug-loaded electrospun poly(L-lactide-co-D/L-lactide) fiber fleeces was investigated on various oral pathogenic bacterial species. At first, the antibacterial effect of fusidic acid in concentrations ranging between 0.062 and 128 μg/mL was observed by means of a microdilution assay. It was shown that all tested species were susceptible toward fusidic acid, with lowest minimal inhibitory concentrations detected for both Actinomycetes spp., *S. aureus*, and *P. gingivalis*.

To date, there is almost no information available about the antibacterial activity of fusidic acid against Actinomyces species. Recent published data revealed an MIC of 0.5 µg/mL fusidic acid for *Actinomyces urogenitalis*, while other authors reported the resistance of one specific strain that was unfortunately not mentioned by name [28,29]. The activity of fusidic acid was also not yet tested against common oral streptococci. As observed in the present investigation, an MIC of 4 µg/mL was detected for most of the Gram-positive strains. This is also in line with data of a recent investigation that proved minimal inhibitory concentrations of 2 and 4 µg/mL fusidic acid against *Enterococcus faecalis* and *Enterococcus faecium* [30]. Generally, most streptococci species seem to be susceptible toward fusidic acid, with MICs ranging between 0.25 and 128 µg/mL, but resistant strains of some clinical isolates have been reported already [31].

Among all tested Gram-negative species, *P. gingivalis* showed the highest susceptibility. It was found that minimal fusidic acid concentrations of 0.062 µg/mL prevented visible bacterial growth. This could be due to the fact that *P. gingivalis* belongs to the genus *Bacteroides*, which are described as being sensitive to fusidic acid [32].

By contrast, minimal inhibitory concentrations of 8 and 32 μg/mL were observed for the Gram-negative species *F. nucleatum* and *A. actinomycetemcommitans*. In the case of *A. actinomycetemcomitans*, other authors reported MICs of fusidic acid that ranged between 0.25 and >256 µg/mL, which was strongly dependent on the respective bacterial subtype [33]. For *F. nucleatum*, there is currently no further information about its susceptibility toward fusidic acid available.

Fusidic acid is primarily directed against common skin pathogens, mainly *S. aureus*, for which it is a potent antibiotic agent [34,35]. Therefore, the species was included in the present study. For S. aureus, an MIC of <0.062 μg/mL was evaluated. As already confirmed by other authors, *S. aureus* is highly susceptibility toward fusidic acid, but with an increasing incidence in global resistance [27,36].

As is known, the antibacterial mechanism of fusidic acid is based on its irreversible binding to the elongation factor G (EF-G) located on the ribosome. Consequently, the translocation of the nascent polypeptide chain from the A site to the P site, the formation of the peptide bond, and the release of the ribosome complex upon reaching the stop codon are all blocked [37,38,39]. Because of its strong interactions with phospholipids, a possible natural resistance of Gram-negative bacteria was suggested as well. This refers the fact that fusidic acid might be captured in the membrane of Gram-negative bacterial cells [40,41].

In order to compare the antibacterial effect of fusidic acid in the present study, metronidazole was used as positive control and showed good antibacterial behavior toward the Gram-negative species *F. nucleatum* (MIC = 0.125 μg/mL) and *P. gingivalis* (MIC = 0.5 μg/mL). Surprisingly, *A. viscosus* (MIC 8 μg/mL) and *A. naeslundii* (MIC 16 μg/mL) were also susceptible. The highest MIC was detected for *A. actinomycetemcomitans* (MIC 64 μg/mL), which is in line with data published by other authors [42].

Primarily, metronidazole presents antibacterial activity against anaerobic bacteria. In the human body, especially the liver, metronidazole is hydroxylated to 2-hydroxymetronidazole and is therefore effective against *A. actinomycetemcommitans* in lower concentrations [43,44,45]. Metronidazole also presents local antibacterial activity. Many approaches already show promising results concerning the local treatment of periodontitis [46,47,48,49,50].

In the present study, the antibacterial activity of monoaxially electrospun polylactide fleeces loaded with fusidic acid in different concentrations was investigated. It was shown that the eluates obtained from theses fleeces efficiently inhibited the growth of *P. gingivalis*, *S. aureus*, *A. viscosus*, and *A. neslundii* over a course of 28 days. In this context, it was found that the diameter of the inhibition zones was larger at the early eluate collection times, which suggests an initial burst release. For this kind of fiber system, burst kinetics are common [51,52]. The effect was also recognized by our own research group with poly(L-lactide-co-D/L-lactide) fleeces loaded with metronidazole in a former study [12]. Other authors, too, also faced this problem. As recently observed with doxycycline-loaded electrospun PLA/HAP nanofibers, 86% of the active ingredient was released during the first 6 h [46]. However, for clinical applications, an initial burst of antibiotics is unfavorable, since a constant drug concentration is required in order to prevent secondary infections.

The process of coaxial electrospinning, on the other hand, enables the manufacturing of fibers with more targeted drug release [11]. In this regard, coaxial nanofibers loaded with fusidic acid have been established already. For those fibers with a core–shell design, a release rate of 66% within 24 h was proven [53].

An ideal drug delivery system is biodegradable, biocompatible, user-friendly, non-irritating to the host, and shows a constant release of the incorporated drug over a defined period of time [13]. Many of those systems have been investigated already. These include fibers, strips, films, microparticles, nanoparticles, gels, membranes, and scaffolds. The duration of constant drug release varies considerably between these systems, with the lowest reported for chlorhexidine-loaded hydroxypropylcellulose films (3 days) [54]. By comparison, while 9 to 18 days were reported for microcycline-loaded nanoparticles, electrospun fibers with incorporated tetracycline showed a constant drug release between 10 and 14 days [13,55,56]. This is also partly consistent with results of the present investigation, in which strong inhibitory effects on *S. aureus*, *P. gingivalis*, and both Actinomyces spp. were observed still after 28 days. A direct comparison of release times is difficult as it depends upon the carrier type, incorporated drug, and intended purpose.

In the present study, the cytotoxicity of fusidic acid and metronidazole was investigated toward gingival fibroblasts too. During the entire study period (48 h), the viability of the exposed cells remained at high levels. For the applied concentrations, no cytotoxic effects were detected, neither for fusidic acid nor for metronidazole. In addition, the eluates collected from the fusidic acid-loaded fleeces did not show any cytotoxic potential over a course of 28 day. This is in line with the results published by Gilchrist et al., who also demonstrated the high cytocompatibility of fusidic acid-loaded electrospun PLGA fibers [57].

One major limitation can be seen in the efficiency of the used fiber system. As shown by previous studies, coaxial fibers with a core–shell design exhibit a more constant drug delivery rate. Therefore, future research should focus on studying the efficiency of coaxial fibers loaded with fusidic acid. For potential clinical use, the choice of polymer is of importance too. In this regard, biodegradable polymers should be favored. In following studies, in vivo efficiency should be investigated as well.

However, the present study proved the good antimicrobial activity of fusidic acid against various oral pathogenic bacteria. In comparison to metronidazole, the effect on *P. gingivalis*, *A. actimomycetemcomitans*, and the *Actinomyces* spp. tended to be even superior.

The eluates obtained from fusidic acid-loaded electrospun fiber fleeces showed inhibitory effects on all Gram-positive cocci species, mostly at the beginning of the observation period, which suggests an initial burst. Strong inhibitory effects of the collected eluates were also proven on *S. aureus*, *A. neslundii*, *A. viscosus*, and *P. gingivalis*. There were no cytotoxic effects detected toward HGFs.

## 5. Conclusions

In the present investigation, a sufficient antibacterial effect of fusidic acid on Gram-negative periodontopathogenic species was observed. Overall, fusidic acid shows great potential in the local treatment of periodontitis. Future studies should focus on the establishment of sufficient coaxial electrospun fiber fleeces that enable a more constant local delivery of fusidic acid. The use of electrospun fiber fleeces loaded with fusidic acid might be attractive for controlling oral pathogens.

## Data Availability

Data can be obtained upon request from the corresponding author.

## Abbreviations

The following abbreviations are used in this manuscript:

AAS	Antibiotic/antimycotic solution
ATCC	American Type Culture Collection
MDPI	Multidisciplinary Digital Publishing Institute
MIC	Minimal inhibitory concentration
DSMZ	Deutsche Sammlung von Mikroorganismen und Zellkulturen
DOAJ	Directory of open access journals
HGF	Human gingival fibroblasts
HPA	Hydroxyapatite
OD	optical density
PLA	Poly lactic acid
PLGA	Poly(lactic-co-glycolic) acid
MRSA	Methicillin-resistant Staphylococcus aureus
SRP	Scaling and root planing
TLA	Three letter acronym
LD	Linear dichroism
